# Suitability and limitations of portion-specific abattoir data as part of an early warning system for emerging diseases of swine in Ontario

**DOI:** 10.1186/1746-6148-8-3

**Published:** 2012-01-06

**Authors:** Andrea L Thomas-Bachli, David L Pearl, Robert M Friendship, Olaf Berke

**Affiliations:** 1 Department of Population Medicine, Ontario Veterinary College, University of Guelph, Guelph, Ontario, Canada; 2 Department of Mathematics and Statistics, University of Guelph, Guelph, Ontario, Canada

## Abstract

**Background:**

Abattoir data have the potential to provide information for geospatial disease surveillance applications, but the quality of the data and utility for detecting disease outbreaks is not well understood. The objectives of this study were to 1) identify non-disease factors that may bias these data for disease surveillance and 2) determine if major disease events that took place during the study period would be captured using multi-level modelling and scan statistics. We analyzed data collected at all provincially-inspected abattoirs in Ontario, Canada during 2001-2007. During these years there were outbreaks of porcine circovirus-associated disease (PCVAD), porcine reproductive and respiratory syndrome (PRRS) and swine influenza that produced widespread disease within the province. Negative binomial models with random intercepts for abattoir, to account for repeated measurements within abattoirs, were created. The relationships between partial carcass condemnation rates for pneumonia and nephritis with year, season, agricultural region, stock price, and abattoir processing capacity were explored. The utility of the spatial scan statistic for detecting clusters of high partial carcass condemnation rates in space, time, and space-time was investigated.

**Results:**

Non-disease factors that were found to be associated with lung and kidney condemnation rates included abattoir processing capacity, agricultural region and season. Yearly trends in predicted condemnation rates varied by agricultural region, and temporal patterns were different for both types of condemnations. Some clusters of high condemnation rates of kidneys with nephritis in time and space-time preceded the timeframe during which case clusters were detected using traditional laboratory data. Yearly kidney condemnation rates related to nephritis lesions in eastern Ontario were most consistent with the trends that were expected in relation to the documented disease outbreaks. Yearly lung condemnation rates did not correspond with the timeframes during which major respiratory disease outbreaks took place.

**Conclusions:**

This study demonstrated that a number of abattoir-related factors require consideration when using abattoir data for quantitative disease surveillance. Data pertaining to lungs condemned for pneumonia did not provide useful information for predicting disease events, while partial carcass condemnations of nephritis were most consistent with expected trends. Techniques that adjust for non-disease factors should be considered when applying cluster detection methods to abattoir data.

## Background

Beginning in late 2004 swine herds in Ontario were affected by outbreaks of severe grower-finisher disease characterized by high mortality and wasting. A major cause of these disease problems was attributed to the emergence of a different strain of porcine circovirus type II (PCV-2), restricted fragment length polymorphism (RFLP) pattern 321 [1]. Post-mortem lesions associated with porcine circovirus-associated disease (PCVAD) in Ontario grower-finisher hogs included bronchointerstitial pneumonia, enterocolitis, and interstitial nephritis [1,2]. Disease losses occurred from the fall of 2004 until late 2006, when effective vaccines to protect against PCV-2 became readily available. Around the same time as the emergence of PCVAD in 2004, an outbreak of a more severe form of porcine reproductive and respiratory virus (PRRSv) took place in southwestern Ontario [3]. This outbreak was closely followed, in the summer of 2005, by the emergence of a triple reassortant subtype H3N2 of swine influenza type A virus (SIV), which swept through Ontario pig herds [4,5]. These large-scale disease events caused severe consequences for swine health in the province and drew attention to the need for strengthened disease surveillance.

Although most infectious diseases of food-producing animals in Canada do not cause disease in humans [6], the recognition that the majority of emerging diseases affecting humans worldwide are zoonotic in origin has raised interest in improving disease surveillance in domestic animals and wildlife [7]. Syndromic surveillance is a relatively new surveillance technique that groups disease cases based on pre-diagnostic syndrome data, rather than specific laboratory diagnoses, to signal a sufficient probability of a case or an outbreak warranting further public or animal health response [8]. The use of pre-diagnostic data sources for syndromic disease surveillance are being considered for their potential to improve timeliness to disease outbreak detection in animal populations [9]. Information from sales-yard [10] and veterinary reports [11], and condemnation data collected at abattoirs [12-14] have recently been investigated for inclusion into animal health syndromic surveillance systems. The Ontario Ministry of Agriculture, Food and Rural Affairs (OMAFRA) meat inspection program utilizes electronic reporting forms as part of the Food Safety Decision-Support System (FSDSS) for condemnation reports and laboratory samples, and could provide near-real-time information for syndromic surveillance. Few published studies to date [12-16] have investigated abattoir data for its potential to provide useful information for disease surveillance, and the validity and reliability of these data for disease surveillance purposes is uncertain. Furthermore, regional differences in farm density and herd management that influence disease prevalence may affect condemnation rates in different geographic areas, and naturally-occurring disease cycles may cause condemnation data to vary seasonally [13,14]. Abattoir processing capacity [13,14] and market value [13] have also been shown to be associated with condemnation rates in provincially-inspected abattoirs in Ontario. Disease outbreak detection requires sensitive and specific quantitative surveillance systems based on knowledge of all relevant predictors and their associations.

From an epidemiological perspective, the study timeframe provided an opportunity to retrospectively investigate the utility of data collected at Ontario provincial abattoirs and the application of spatial scan statistics for detecting clusters of high partial carcass condemnation rates. We hypothesized that yearly patterns for kidney and lung condemnations would reflect the years during which increased respiratory disease and nephritis affected swine herds across Ontario. Similar clusters were expected to be detected in space, time, and space-time, for both partial carcass condemnation reasons. We were particularly interested in determining whether clusters could be detected earlier than those found with traditional passive surveillance using laboratory data.

Based on discussions with staff at OMAFRA, there are questions regarding the quality of data recording for the condemnation of organs that are not considered to be economically important or a concern for food safety. As with any disease surveillance system, the quality of the data to be analyzed is of foremost concern [17]. Consequently, this study has the following objectives: i) examine the utility of partial carcass condemnation data from provincially inspected abattoirs in the province of Ontario, Canada, for inclusion in a syndromic surveillance system for finisher pigs; ii) explore potential sources of bias in the data that are unrelated to swine health status; and iii) identify gaps in the data where types of condemnation lesions were never reported.

## Methods

### Data sources and database management

**Figure 1:**
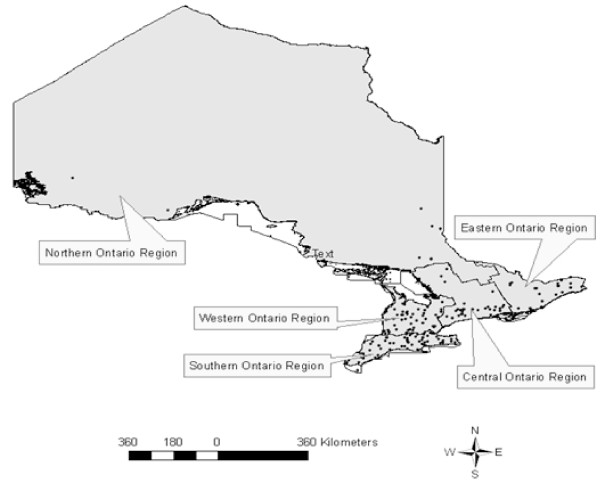
**Ontario Provincial abattoirs which processed market hogs between 2001-2007 and census agricultural region**.

Partial carcass condemnation data were obtained with permission from the Ontario Ministry of Agriculture, Food and Rural Affairs (OMAFRA) Food Safety Decision Support System (FSDSS) database. The database contained the daily numbers of market hogs slaughtered, condemned, and the partial carcass condemnation reasons documented by provincial meat-inspectors in all provincially-inspected abattoirs across the province of Ontario, Canada from 2001-2007. There were 34 partial carcass condemnations categories, of which we investigated condemnations due to pneumonia lung lesions and kidney nephritis lesions. Geographic coordinates for each abattoir were used to create a digital layer of point locations over a map of Ontario in a Canada Lambert Conformal Conic projection using ArcGIS 9.2 (ESRI, Redlands, CA, USA) (Figure 1).

A layer on the map was created for each of five census agricultural regions (CARs) in the province, central, eastern, western, northern and southern Ontario, using a geographic shape file from Statistics Canada [18] (Figure 1). The abattoir locations were linked with their corresponding agricultural regions and added to the dataset using a point in polygon procedure in ArcGIS 9.2. Historical weekly market hog stock prices were compiled from Agriculture Canada [19]. Data were merged into a common dataset using Stata 10.0 (StataCorp, College Station, TX, USA). Variables for quarterly median hog stock price, quarterly number of hogs processed and condemned, and quarterly lung condemnations at each abattoir were developed. A variable representing season was created with the following 3 month categories: winter (Jan-Mar), spring (Apr-June), summer (July-Sept), and autumn (Oct-Dec). A variable was created for the total number of hogs processed per year at each facility by adding the number of hogs slaughtered and condemned at each abattoir per year. The number of weeks each abattoir processed market hogs was created by summing the number of weeks each abattoir processed at least one market hog during each year of the study.

### Statistical models

Statistical analyses were performed using STATA Intercooled 10.0 (StataCorp, College Station, Texas). The total number of lungs condemned per quarter and the number of kidneys condemned for nephritis were our dependant variables, and the following variables were examined for their association with lung and kidney condemnation rates: year, season, census agricultural region, median quarterly market hog stock price, number of weeks each abattoir processed swine carcasses per year, and the total number of market hogs processed per year. Linearity between outcome variables and continuous predictors were assessed using locally-weighted regression scatterplot smoothing (lowess) curves [20]. Any continuous variables found to have a non-linear relationship with these condemnation rates were categorized, if an appropriate transformation could not be found or the relationship could not be modelled with the addition of a quadratic term [20]. Spearman's rank correlation coefficients (r_s_) were examined for each pair of independant variables to assess collinearity. If any pair of variables was found to be strongly correlated, using a cut-point of r_s _≥ 0.8, the variable considered most informative from a biological perspective was included in the proceeding models [20].

Poisson and negative binomial models with random intercepts for abattoir, to account for repeated measurements within abattoirs, were created. Random effects Poisson regression models were fit using an adaptive quadrature technique [21] as implemented in STATA using its xtmepoisson command. Random intercept negative binomial models were used to accommodate overdispersion due to clustering at abattoirs; the models included beta distributed random effects and were fit in STATA using the xtnbreg command [22]. The offset for each model was the log_10 _number of hogs processed per quarter and the outcome was either the number of lungs condemned due to pneumonia, or the number of kidneys condemned for nephritis, per quarter. Univariable statistical analysis was performed to assess the association of each variable with quarterly lung condemnation rate and quarterly kidney condemnation rate. Any variables with a liberal significance level alpha = 0.20 were included for multi-variable modelling and a backward step-wise approach was used to create the final main effects multivariable model. Likelihood ratio tests were used to determine the significance of each variable in addition to evaluation of nested models with and without interaction terms that were deemed to be biologically plausible [20]. The interactions tested were as follows: year and CAR, year and season, and CAR and season. Confounding was assessed by evaluating the removal of each non-significant variable from the model. A variable was considered a confounder if it was not an intervening variable, and if its removal resulted in a 20% or greater change in any of the coefficients of statistically significant variables [20]. All tests were two-tailed and variables with a significance level alpha = 0.05 were considered statistically significant. The coefficients were exponentiated and reported as incident rate ratios (IRRs). For covariate combinations where the case count was 0, median unbiased estimates and Poisson confidence intervals were estimated to give an approximation of the IRR and 95% confidence interval. The lincom statement in STATA was used to create contrasts between the predicted outcomes and interaction terms. To assess the best-fitting model between the negative binomial and Poisson models with random intercepts for abattoir, we used Akaike's Information Criterion (AIC) and Bayesian Information Criterion (BIC). The model with the lowest AIC and BIC was considered the best fitting model [20]. To assess outliers and evaluate model fit, depending upon the options available for a particular model, we examined crude, Pearson, Anscombe and deviance residuals.

### Retrospective scan statistics

Latitude and longitude coordinates and daily numbers of lung condemnations due to pneumonia and kidney condemnations due to nephritis for each provincially-inspected abattoir in Ontario were used to detect spatial, temporal and spatiotemporal clusters of high lung and kidney condemnation rates. The data were evaluated for clustering during the entire study period (2001-2007) using SaTScan version 8.0 (http://www.satscan.org). The scan statistic was used to compare the number of observed to expected lung and kidney condemnations following a random distribution, by imposing windows that vary in space, time, or three-dimensional space-time [22,23]. The model distribution for the spatial scan tests was a Poisson model, which uses case and population data to compare rates of condemnation in the windows to the underlying population [22,23]. A Bernoulli model was used for the temporal scan tests. This distribution avoids issues with biased cluster detection that may have occurred as a result of timeframes in the data when abattoirs were closed, because it compares cases with a control dataset comprised of the number of healthy animals slaughtered [22]. The space-time scan tests require only case data containing the abattoir identification and the date of condemnation. The case and population datasets contained the number of lungs or kidneys condemned and the daily number of hogs processed at each abattoir location, respectively, by provincial inspectors. Maximum scanning windows were limited to 50% of the population of hogs slaughtered, or population at risk (PAR) for the spatial scans, 50% of the study period for temporal scan tests, and both 50% of the condemnations and study period for the space-time scan tests. A scanning time aggregation length of 1 day was specified for the temporal and space-time scan tests. To determine the significance level, 9999 Monte Carlo replications were run for the spatial and temporal scan tests, and 999 replications were run for the space-time scans to avoid excessive computation time. For the spatial scans, we reported only those clusters that had no spatial overlap. Reporting of overlapping clusters for the space-time scans was permitted, as long as there were no pairs of centres in each others clusters. This allowed clusters that overlapped in space but took place during different time periods to be captured. Only non-overlapping clusters in space-time were reported. We compared results from our scans qualitatively with the literature [1,2,4] and historical reports from the Animal Health Laboratory (AHL) [3,5], which is the primary location where swine practitioners in Ontario submit samples for diagnostics.

### Non-reporting bias

We also investigated whether there was evidence of spatial clusters of abattoirs which did not report lung pneumonia or kidney nephritis condemnations. We used a Bernoulli model, with cases as the abattoirs where these organs were not recorded as condemned, and controls as all other abattoirs in the province. Settings were otherwise the same as preceding spatial scans.

## Results

### Descriptive statistics

The number of provincially-inspected abattoirs in Ontario declined each year from 154 in 2001 to 106 in 2007, and each premise was present during the 7 year study period for an average of 4.2 years (95% CI: 3.9-4.6). There were 2,264,409 market hogs slaughtered in provincially-inspected abattoirs in Ontario during 2001-2007, and 13,206 whole hogs were condemned as unfit for human consumption. A total of 649,106 partial carcasses were condemned, for a rate of 28.67 partial carcasses condemned per 100 hogs slaughtered. Liver parasites were the most common partial condemnation category [see Additional file 1, Table S1], accounting for almost 60% of all partial condemnations. Other partial condemnation categories that were most frequently listed by meat inspectors were heart adhesions, liver adhesions, cystic kidneys, pneumonic lungs, elbow/hock arthritis, and kidneys due to nephritis [see Additional file 1, Table S1].

**Figure 2:**
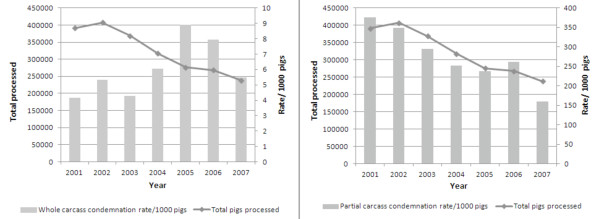
**Partial carcass and whole carcass condemnation rates by year, and total number of market hogs processed per year**.

**Figure 3:**
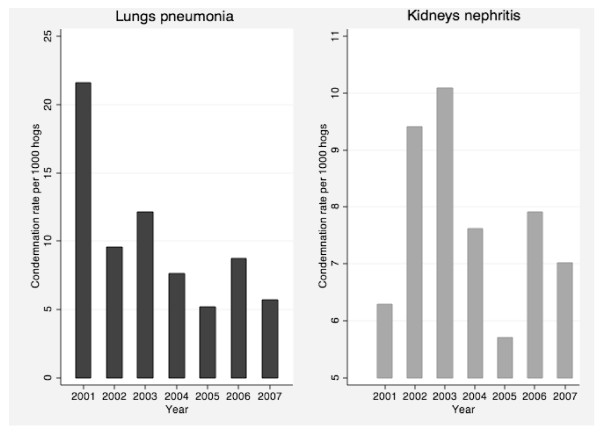
**Partial carcass condemnation rates for lungs with pneumonia lesions and kidneys with nephritis lesions**.

Over the course of the study period, there was a declining trend in partial condemnation rates (Figure 2a), whereas whole carcass condemnations peaked during 2004 and 2005 (Figure 2b). When we examined partial condemnation rates for lung condemnations, we found that the rates of lung condemnations were much higher in 2001 compared with all subsequent years, and there was no notable trend in rates over the remainder of the study period (Figure 3).

Partial condemnations related to kidneys with nephritis lesions were highest between 2002-2003 and lowest in 2005 (Figure 3). By census agricultural region, the condemnation rate for lungs was highest in the Western region, and lowest in Northern Ontario (Table 1). In contrast, the condemnation rates for kidneys due to nephritis lesions were highest in Central Ontario and lowest in Southern Ontario regions (Table 2). Condemnation rates for lungs showed a slight decreasing trend through the seasons from winter to fall, with rates highest in the winter and lowest in the fall (Table 1). Kidney condemnations due to nephritis lesions did not follow any clear seasonal trend (Table 2).

**Table 1:** Number of lungs condemned and condemnation rates by census agricultural region (CAR) and season

CAR	# of lungs condemned/# pigs processed	Lung condemnation rate per 1000 pigs processed	Season	# of lungs condemned/# pigs processed	Lung condemnation rate per 1000 pigs processed
**Central Ont**.	1232/120 424	10.23	**Winter**	7149/567 397	12.60

**Eastern Ont**.	334/59 798	5.59	**Spring**	6652/561 568	11.85

**Northern Ont**.	17/21 701	0.78	**Summer**	5516/557 768	9.89

**Southern Ont**.	5883/1 141 959	5.15	**Fall**	5062/577 676	8.76

**Western Ont**.	16913/920 527	18.37			

Number of lungs condemned and condemnation rates by census agricultural region (CAR) and season in market hogs (pigs) in provincially-inspected abattoirs in Ontario during 2001-2007.

**Table 2:** Number of kidneys condemned and condemnation rates by census agricultural region (CAR) and season

CAR	Number of kidneys condemned/pigs processed	Kidney nephritis regional condemnation rate/1000 pigs processed	Season	Number of kidneys condemned/pigs processed	Kidneys nephritis seasonal condemn-ation rate/1000 pigs processed
**Central Ont**.	6513/120 424	54.08	**Winter**	4583/567 397	8.08
**Eastern Ont**.	969/59 798	16.20	**Spring**	4413/561 568	7.86
**Northern Ont**.	241/21 701	11.11	**Summer**	4083/557 768	7.32
**Southern Ont**.	1605/1 141 959	1.41	**Fall**	4711/577 676	8.16
**Western Ont**.	8462/920 527	9.19			

Number of kidneys condemned for nephritis and condemnation rates by census agricultural region (CAR) and season in market hogs (pigs) in provincially-inspected abattoirs in Ontario during 2001-2007.

### Statistical models

Negative binomial regression models with a random intercept for abattoir were generally outperforming the random intercept Poisson regression models, with respect to the AIC and BIC. The results of the negative binomial models are as follows:

### Univariable models

The variables for the number of weeks an abattoir processed hogs per year and the total number of hogs processed per year were found to be highly correlated (r_s _= 0.81), so we chose to build the models using the variable for total hogs processed per year as a measure of abattoir capacity. This variable was modelled on a logarithmic scale for the lung condemnation model in order to linearize the relationship with the outcome. No other combination of variables demonstrated correlation greater than r_s _= 0.16. The results of our univariable negative binomial models with a random intercept for abattoirs demonstrated the following, based on a Wald test [see Additional File 2, Table S2]: In comparison with 2001, all subsequent years had significantly lower lung condemnation rates, whereas 2002 and 2003 showed significantly higher kidney nephritis condemnation rates in comparison with 2001, and 2007 was associated with significantly lower condemnation rates of kidney nephritis lesions [see Additional File 2, Table S2]. In comparison with winter, summer and fall were associated with reduced lung and kidney condemnation rates and spring was associated with a reduced kidney condemnation rate compared to winter [see Additional File 2, Table S2]. We found differing regional variation in condemnation rates for kidney nephritis lesions and lungs classified with pneumonia [see Additional File 2, Table S2]. We also found that for each dollar increase in quarterly hog stock price, there was a slightly higher lung condemnation rate observed [see Additional File 2, Table S2], but there was no significant association between hog stock prices and kidney nephritis condemnation rates [see Additional File 2, Table S2]. There was a consistent trend observed whereby abattoirs with larger processing capacities were associated with a sparing effect on condemnation rates for both organ condemnation categories [see Additional File 2, Table S2].

### Multivariable models

When all significant variables from our multivariable models were considered, year, log_10 _total number of hogs processed per year, and an interaction between year and census agricultural region were significantly associated with partial condemnation rates for lung condemnations in our multivariable model (Table 3). Year, the number of hogs processed per year, an interaction between census agricultural region and season, and an interaction between census agricultural region and year were significantly associated with condemnation rate for kidneys due to nephritis in our multivariable model. There was no evidence for confounding from any excluded variables removed from either model.

**Table 3:** Multivariable random intercept negative binomial models

	Lungs Pneumonia			Kidneys Nephritis		
***Variable***	***IRR***	***P-value***	***95% CI***	***IRR***	***P-value***	***95% CI***

**Year**						
**2001**	referent			referent		
**2002**	1.10	0.76	0.69-1.98	1.53	0.02	1.06-2.20
**2003**	0.96	0.90	0.50-1.83	1.34	0.15	0.90-1.98
**2004**	0.28	0.004	0.12-0.66	1.06	0.78	0.70-1.60
**2005**	0.23	0.001	0.09-0.56	0.96	0.83	0.63-1.45
**2006**	0.11	< 0.001	0.03-0.39	1.28	0.23	0.86-1.92
**2007**	0.08	0.001	0.02-0.33	0.99	0.98	0.65-1.53
**Agricultural region**						
**Central**	referent			referent		
**Eastern**	1.16	0.67	0.59-2.32	0.88	0.67	0.48-1.61
**Northern**	0.15	0.08	0.02-1.22	0.67	0.51	0.20-2.20
**Southern**	0.54	0.04	0.30-0.96	0.06	< 0.001	0.03-0.12
**Western**	0.97	0.92	0.53-1.77	0.08	< 0.001	0.04-0.16
**Season**						
**Winter**	referent			referent		
**Spring**	N/A			1.08	0.56	0.84-1.39
**Summer**	N/A			0.98	0.90	0.76-1.28
**Fall**	N/A			0.93	0.54	0.72-1.19
# Pigs processed/year*	0.50	< 0.001	0.46-0.54	0.99995	< 0.001	0.99995-0.99996
	
	**Pneumonia**			**Nephritis**		
	
	***IRR***	***P-value***	***95% CI***	***IRR***	***P-value***	***95% CI***

**Year × Agricultural Region**						
**2002 × Eastern Ont**.	0.32	0.02	0.12-0.82	0.72	0.31	0.38-1.35
**2002 × Northern Ont**.	0.15	0.08	0.02-1.22	1.23	0.73	0.37-4.14
**2002 × Southern Ont**.	0.48	0.05	0.23-1.00	1.94	0.08	0.93-4.02
**2002 × Western Ont**.	0.77	0.49	0.36-1.62	1.02	0.96	0.53-1.96
**2003 × Eastern Ont**.	0.34	0.04	0.12-0.94	0.43	0.03	0.21-0.91
**2003 × Northern Ont**.	3.45	0.30	0.33-36.38	1.81	0.34	0.54-6.04
**2003 × Southern Ont**.	0.28	0.004	0.12-0.67	2.38	0.03	1.12-5.08
**2003 × Western Ont**.	0.65	0.30	0.29-1.46	1.26	0.50	0.64-2.47
**2004 × Eastern Ont**.	0.56	0.41	0.14-2.22	0.93	0.85	0.45-1.94
2004 × Northern Ont.*	0.0002	1.00	0.0-0.0009	2.54	0.14	0.75-8.60
**2004 × Southern Ont**.	0.98	0.97	0.34-2.83	3.23	0.01	1.50-6.95
**2004 × Western Ont**.	2.48	0.08	0.91-6.77	0.85	0.67	0.39-1.84
**2005 × Eastern Ont**.	1.11	0.88	0.30-4.03	1.88	0.07	0.95-3.72
2005 × Northern Ont.*	0.0002	1.00	0.0-0.001	0.91	0.90	0.19-4.23
**2005 × Southern Ont**.	0.59	0.41	0.17-2.06	4.07	< 0.001	1.85-8.93
**2005 × Western Ont**.	1.61	0.40	0.53-4.89	0.82	0.64	0.36-1.88
**2006 × Eastern Ont**.	2.06	0.37	0.43-9.90	1.86	0.11	0.86-4.01
2006 × Northern Ont.*	0.0003	1.00	0.0-0.0014	1.19	0.82	0.28-5.02
**2006 × Southern Ont**.	3.17	0.10	0.81-12.35	2.90	0.01	1.32-6.34
**2006 × Western Ont**.	3.95	0.05	1.02-15.35	0.81	0.61	0.37-1.79
2007 × Eastern Ont.*	0.0001	1.00	0.0-0.0005	1.80	0.10	0.89-3.66
2007 × Northern Ont.*	0.0004	1.00	0.0-0.0018	2.51	0.21	0.60-10.59
**2007 × Southern Ont**.	4.54	0.08	0.93-22.21	5.70	< 0.001	2.64-12.32
**2007 × Western Ont**.	4.64	0.06	0.95-22.68	1.86	0.11	0.86-4.01

**Kidneys nephritis model**						
			
	**IRR**	**P-value**	**95% CI**			
			
**Season × census agricultural region**						
**Winter × Central Ont**.	referent					
**Spring × Eastern Ont**.	1.02	0.92	0.64-1.63			
**Summer × Eastern Ont**.	0.81	0.39	0.50-1.31			
**Fall × Eastern Ont**.	0.92	0.72	0.58-1.46			
**Spring × Northern Ont**.	0.25	0.01	0.09-0.72			
**Summer × Northern Ont**.	0.34	0.02	0.13-0.87			
**Fall × Northern Ont**.	0.75	0.46	0.35-1.60			
**Spring × Southern Ont**.	0.78	0.31	0.48-1.27			
**Summer × Southern Ont**.	1.02	0.93	0.63-1.65			
**Fall × Southern Ont**.	0.77	0.31	0.47-1.27			
**Spring × Western Ont**.	1.65	0.08	0.94-2.90			
**Summer × Western Ont**.	1.54	0.15	0.86-2.79			
**Fall × Western Ont**.	1.85	0.03	1.05-3.24			

Modeling the association between lung pneumonia and kidney nephritis condemnation rates in provincial abattoirs in Ontario and year, season, census agricultural region, total pigs processed per year and median quarterly hog stock price, with abattoir as a random effect

**Figure 4:**
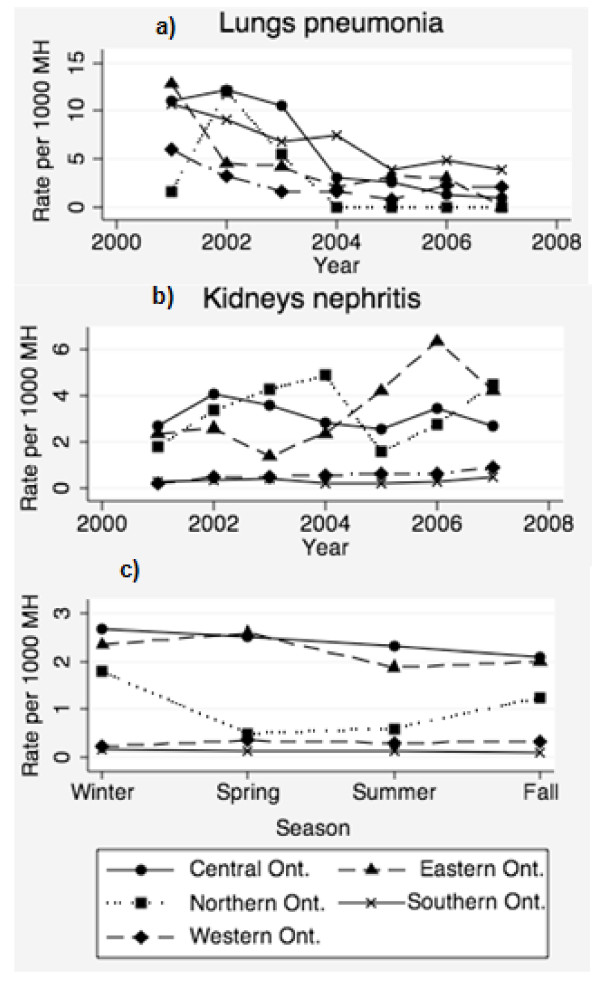
**Graphical representation of the predicted values for the interactions from the multivariable models**. Predicted values were estimated from the final fitted random effects negative binomial model for a) lung condemnations due to pneumonia and b) kidney condemnations due to nephritis, and the interaction between season and census agricultural region for the kidney nephritis model c), with the remaining variables set to their referent values.

We found that when all other variables were considered, for every unit increase in the number of hogs processed per year by an abattoir (measured as a log_10 _transformation for the lungs model), there was a significant decrease in both lung and kidney condemnation rates (Table 3). Upon examination of the predicted rates for lung condemnations by year and agricultural region, in general we observed a decline in condemnation rates throughout the years of the study period (Figure 4a). Except for northern and central Ontario, which peaked in 2002, all other regions showed the highest lung condemnations in 2001 (Figure 4a). When we examined the predicted rates for kidney condemnations due to nephritis by each year and agricultural region, we observed that kidney condemnations were relatively low for all years in southern and western Ontario compared to other regions. Rates peaked during 2002 in central Ontario, 2004 and 2007 in northern Ontario, and 2006 in eastern Ontario (Figure 4b). A comparison of the predicted kidney condemnation rates for each region by season revealed the most seasonal variation in northern Ontario, with the highest rates observed in winter and fall compared to spring and summer (Figure 4c). Predicted rates in the other regions of the study did not vary greatly among the seasons (Figure 4c).

### Retrospective scan statistics

#### Spatial scan tests

**Figure 5:**
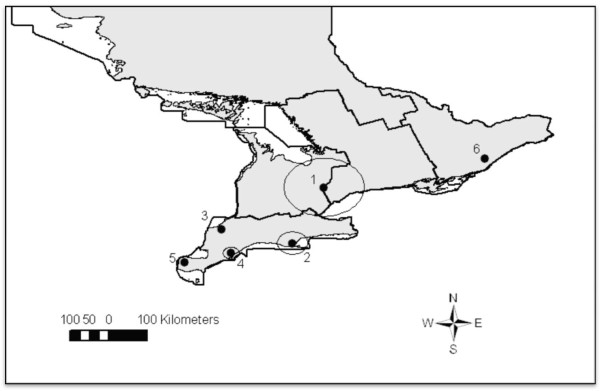
**Spatial clusters of high lung condemnation rates due to pneumonia in Ontario provincial abattoirs (2001-2007)**. Satscan settings were specified as Poisson model with 50% of the population at risk (total market hogs slaughtered) as the maximum spatial scanning window. Cluster centroids are indicated by solid dots and boundaries are indicated by circles, numbered in descending order from the most likely to least likely.

**Figure 6:**
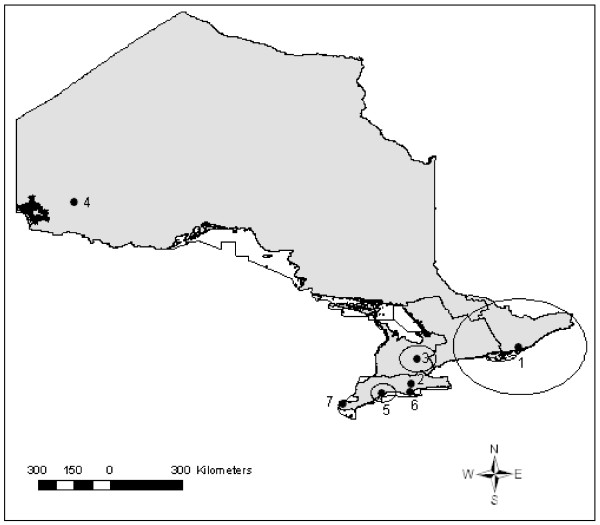
**Spatial clusters of high kidney condemnation rates due to nephritis in Ontario provincial abattoirs (2001-2007)**. Satscan settings were specified as Poisson model with 50% of the population at risk (total market hogs slaughtered) as the maximum spatial scanning window. Cluster centroids are indicated by solid dots and boundaries are indicated by circles, numbered in descending order from the most likely to least likely.

**Figure 7:**
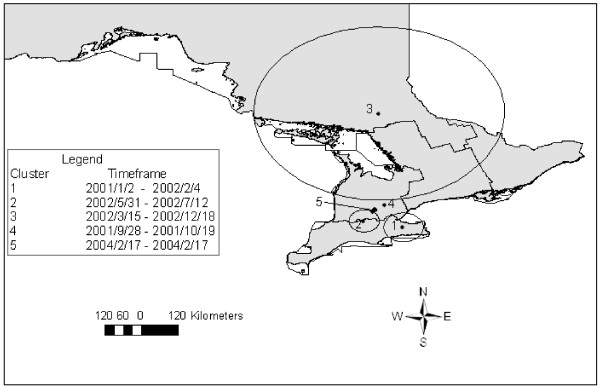
**Space-time clusters of high lung condemnation rates due to pneumonia in Ontario provincial abattoirs (2001-2007)**. Satscan settings were specified as: Space-time permutation model with 50% of the population at risk specified as the maximum spatial scanning window, 50% of the study period set as the maximum temporal scanning window and 1 day set as the maximum time precision. Cluster centroids are indicated by solid dots and boundaries are indicated by circles, numbered in descending order from most likely to least likely.

There were 6 statistically significant (p < 0.05) non-overlapping clusters of high lung condemnations detected when the entire study period was scanned spatially, including 3 clusters containing single abattoirs (Table 4, Figure 5). The most likely cluster was located in western, central and a small portion of southern Ontario and contained 36 abattoirs (Figure 5). The other significant clusters were all located in southern Ontario except for the sixth most likely cluster, which was located in eastern Ontario and contained a single abattoir (Figure 5). When we employed the spatial scan statistic to detect clusters of high kidney condemnations due to nephritis, the most likely spatial cluster was found to include most of eastern and a large portion of central Ontario regions (Table 5, Figure 6). The remaining smaller clusters were located in southern and western Ontario, except for a cluster containing a single abattoir in northern Ontario region (Figure 7).

**Table 4:** Summary of spatial clusters of high lung condemnations

Cluster	# Abattoirs	Radius (km)	# Lungs	# Pigs	CR/1000 pigs	RR	P-value
**1**	36	75.43	15 444	706 969	21.85	3.81	0.0001
**2**	5	29.16	4 318	64 270	67.19	7.37	0.0001
**3**	1	0	135	3 790	35.62	3.32	0.0001
**4**	2	15.46	130	4 967	26.17	2.44	0.0001
**5**	1	0	99	3 692	26.82	2.50	0.0001
**6**	1	0	197	12 981	15.18	1.41	0.0027

Summary of statistically significant (p < 0.05) spatial clusters* of high lung condemnations for Ontario Provincial abattoirs (2001-2007).**

*SaTScan settings specified as: maximum scanning window of 50% of market hogs (pigs) slaughtered.

** Descriptive statistics indicating total lungs condemned, carcasses processed (# pigs), corresponding condemnation rates (CR) per 1000 market hogs (pigs) processed and relative risks (RR).

**Table 5:** Summary of spatial clusters of high kidney condemnations

Cluster	# Abattoirs	Radius (km)	# Kidneys	# Pigs	CR/1000 pigs	RR	P-value
**1**	51	198.41	7 202	148 728	48.42	9.68	0.0001
**2**	22	55.17	8 211	747 652	10.98	1.74	0.0001
**3**	1	0	291	5 178	56.20	7.26	0.0001
**4**	1	0	142	1 923	73.84	9.47	0.0001
**5**	5	37.91	236	12 905	18.29	2.35	0.0001
**6**	1	0	175	9 051	19.33	2.48	0.0001
**7**	1	0	20	336	59.52	7.58	0.0001

Summary of statistically significant (p < 0.05) spatial clusters* of high kidney condemnations for Ontario Provincial abattoirs (2001-2007).**

*SaTScan settings specified as: maximum scanning window of 50% of market hogs (pigs) slaughtered.

** Descriptive statistics indicating total kidneys condemned, carcasses processed (# pigs), corresponding condemnation rates (CR) per 1000 market hogs (pigs) processed and relative risks (RR).

#### Space-time scan tests

**Figure 8:**
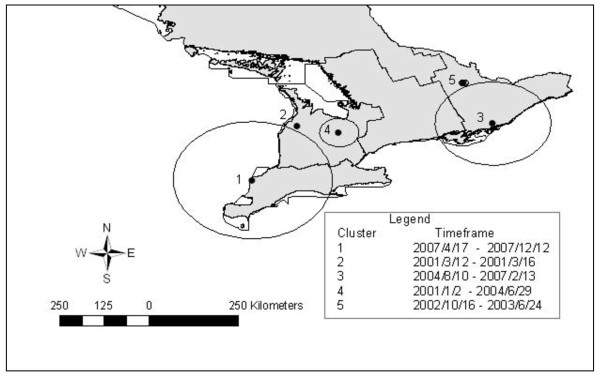
**Space-time clusters of high kidney condemnation rates due to nephritis in Ontario provincial abattoirs (2001-2007)**. Satscan settings were specified as: Space-time permutation model with 50% of the population at risk specified as the maximum spatial scanning window, 50% of the study period set as the maximum temporal scanning window and 1 day set as the maximum time precision. Cluster centroids are indicated by solid dots and boundaries are indicated by circles, numbered in descending order from most likely to least likely.

Space-time scans of lung condemnations during the entire study period revealed five statistically significant clusters (Table 6, Figure 7). The most likely cluster was comprised of 16 abattoirs in the eastern part of southern Ontario, during early 2001 until early 2002 (Table 6, Figure 7). Space-time clusters of high lung condemnations were detected during only 2001 and 2002, except for the fifth most likely cluster which took place in 2004 over a single day (Table 6). All seasons of the year were represented in these clusters (Table 6). High kidney condemnations due to nephritis were also found to cluster in space-time. The most likely cluster contained abattoirs in southern and western Ontario, and took place towards the end of the study period (Table 7, Figure 8). The remaining space-time clusters of high kidney condemnation rates took place in 2001 and 2002, with the exception of the third most likely cluster, which occurred between 2004/8/10-2007/2/13 and contained a large region of 21 abattoirs in eastern and central Ontario (Table 7, Figure 8). Clusters of kidney condemnations due to nephritis in space-time ranged in size from a single abattoir to 163.92 km, and included all seasons (Table 7).

**Table 6:** Summary of space-time clusters of high lung condemnation rates

Cluster	# of abattoirs	Radius (km)	Timeframe	# of lungs condemned/pigs	CR/1000 pigs	O/E
**1**	16	47.72	2001/1/2-2002/2/4	4 096/74 397	55.06	2.32
**2**	22	36.47	2002/5/31-2002/7/12	130/2 203	54.47	53.67
**3**	61	285.10	2002/3/15-2002/12/18	669/40 736	16.42	3.20
**4**	1	0	2001/9/28-2001/10/19	14/23	608.70	77.64
**5**	1	0	2004/2/17-2004/2/17	5/116	43.10	2708.78

Summary of statistically significant (p < 0.05) space-time clusters* of high partial carcass condemnation rates in Ontario Provincial abattoirs for lungs (2001-2007).**

* SaTScan settings: maximum spatial scanning window specified as 50% of cases; maximum temporal scanning window specified as 50% of the study period.

** Descriptive statistics indicating number of abattoirs in each cluster, cluster radius, cluster timeframe, total lungs condemned per number of market hogs processed (pigs), corresponding condemnation rate per 1000 pigs and observed/expected (O/E) ratio.

**Table 7:** Summary of space-time clusters of high kidney condemnation rates

Cluster	# of abattoirs	Radius (km)	Timeframe	# of kidneys condemned/pigs	CR/1000 pigs	O/E
**1**	51	163.92	2007/4/17-2007/12/12	626/18 958	33.02	4.49
**2**	1	0	2001/3/12-2001/3/16	80/714	112.04	115.85
**3**	21	116.45	2004/8/10-2007/2/13	2393/39 747	60.21	39.74
**4**	11	39.74	2001/1/2-2004/6/29	5725/254 879	22.46	1.30
**5**	3	8.85	2002/10/16-2003/6/24	395/1 581	249.84	3.65

Summary of statistically significant (p < 0.05) space-time clusters* of high partial carcass condemnation rates in Ontario Provincial abattoirs for kidneys (2001-2007).**

* SaTScan settings: maximum spatial scanning window specified as 50% of cases; maximum temporal scanning window specified as 50% of the study period.

** Descriptive statistics indicating number of abattoirs in each cluster, cluster radius, cluster timeframe, total kidneys condemned per number of market hogs processed (pigs), corresponding condemnation rate per 1000 pigs and observed/expected (O/E) ratio.

#### Temporal scan tests

Temporal scans run for the entire study period, with 50% of the study duration as the maximum temporal window, showed the most likely cluster of high lung condemnation rates took place from 2001/1/8-2001/9/11 (p = 0.0001) and the most likely cluster of high kidney condemnation rates occurred during 2001/10/30-2004/2/24 (p = 0.0001) (Table 8).

**Table 8:** Summary of temporal clusters of high condemnation rates in Ontario Provincial abattoirs (2001-2007)

Partial condemnation reason	Timeframe	# Organ condemnations/# pigs processed	Condemnation rate/1000 pigs processed	RR
**Lungs pneumonia**	2001/1/8-2001/9/11	6786/270965	25.05	2.94
**Kidneys nephritis**	2001/10/30-2004/2/24	8829/914963	9.65	1.48

Summary of statistically significant (p < 0.05) temporal clusters* of high partial carcass condemnation rates in Ontario Provincial abattoirs (2001-2007).**

Descriptive statistics indicating total lungs and kidneys condemned/total number of pigs processed, corresponding condemnation rates per 1000 pigs processed and relative risks

* SaTScan settings specified as maximum scanning window of 50% the study period.

** Descriptive statistics indicating total lungs and kidneys condemned/total number of pigs processed, corresponding condemnation rates per 1000 pigs processed and relative risks (RR).

### Non-reporting bias

We scanned spatially for clusters of low reporting of lung condemnations, and found a single cluster containing 26 abattoirs in southern Ontario which did not report lung condemnations during the study period (radius = 92.14 km, p = 0.0001). There was no evidence of non-reporting for kidney condemnations.

## Discussion

In this study, we explored non-disease factors that affect partial carcass condemnations at provincial abattoirs across Ontario. An advantage of this study was that historical data were available during a time when large-scale disease events took place for performance evaluation. We used multivariable statistical models that included a random intercept for repeated observations from abattoirs to explore the relationships between temporal, regional, seasonal and abattoir-related factors for both lung and kidney condemnations. We also evaluated spatial, temporal and space-time clusters of high rates of kidney and lung partial carcass condemnations in reference to the widespread outbreaks of PCVAD, PRRSv and swine influenza virus that took place.

Pneumonia is one of the most frequently seen clinical features of PCVAD, and broncho-interstitial pneumonia was a common pathological feature of PCV-2 cases seen at the AHL during the outbreak [1]. Nephritis was another pathological feature of the PCVAD outbreak cases [1,24], can also be caused by PRRSv infection [25,26], and is easily visualized on post-mortem inspection. We expected that these two condemnation categories would be good indicators for the detection of the large-scale outbreaks of PCVAD, PRRS and swine influenza. Lung condemnations due to pneumonia and kidney condemnations due to nephritis were both prevalent causes for partial condemnations of hogs in provincial abattoirs during 2001-2007 (Additional file 1, Table S1). Results from our negative binomial models and scan statistics revealed that lung condemnation data were not useful for capturing trends in condemnations related to these outbreaks. In contrast, some of the results from our models and scan statistics using the data pertaining to kidney condemnations in eastern Ontario mirrored expected findings during the timeframe when the PCV-2 outbreak occurred. Of note, one space-time cluster of kidney condemnations took place earlier than a cluster of PCV-2 cases detected using traditional lab data [27]. The space-time permutation model controls for purely spatial clusters, which is important because of the regional differences in lung and kidney condemnations detected by our models. Model-adjusted space-time scan statistics may further improve upon cluster detection [28,29]. To determine whether space-time clusters are the result of true disease outbreaks, validation with other information, such as source farm and laboratory records would be required before a formal prospective system is introduced.

Marked differences in condemnation rates for pigs with pneumonia and partial carcass condemnations are found between abattoirs in other countries with well-developed meat inspection, including Belgium [30], Denmark [31] and Italy [32]. A study in Denmark also demonstrated large variability in the sensitivity and specificity of traditional inspection related to chronic pleuritis lesions between 4 abattoirs that was greatly improved by more detailed, standardized post-mortem inspection [32]. It is possible that our data were affected by variations in the sensitivity and specificity of lesion detection between individual abattoirs and over time. Our space-time scans should have adjusted for purely spatial clusters. Standardized recording, such as described for scoring lung lesions [32,33] could also be employed by abattoirs to reduce biases related to variation of lesion recording. This would likely result in some cost to timeliness of data collection and would require enhanced training of meat inspectors.

A feature of provincially-inspected abattoirs in Ontario that differs from abattoirs in a number of other countries is that a lay meat inspector may partially condemn pig carcasses in provincial abattoirs in the absence of direct veterinary oversight [34]. Provincial meat inspectors undergo a rigorous classroom and field training program, and efforts are underway to improve standardization of inspection procedures across the province [35]. Standard guidelines are used for classifying pathological lesions for partial and whole carcass condemnations according to the Canadian Food Inspection Agency's Meat Hygiene Manual of Procedures [36]. However, there may be greater variability in the classification of partial condemnation lesions among non-veterinary inspectors with less specialized training in pathology, anatomy and specific disease conditions, leading to increased recording bias between inspectors and individual abattoirs. Regardless of jurisdictional meat inspection training requirements, the subjective nature of meat inspection makes observational and operator bias a concern, especially when combining data from multiple slaughterplants for spatial and space-time surveillance applications.

An association between larger abattoirs and lower condemnation rates for both lung and kidney condemnations agreed with findings that demonstrated the same effect for whole carcass condemnation rates [14]. One plausible explanation to explain this finding is that pigs being shipped to larger abattoirs have higher health status. Another reason behind this association might be the effect of faster processing speeds in large abattoirs on the ability of inspectors to thoroughly examine a carcass and/or record lesions. Unfortunately, we did not have access to processing line speeds or source farm information to explore these relationships.

There were no significant seasonal trends in partial condemnation rates related to lung lesions, which is inconsistent with previous research documenting a higher rate of lesions consistent with pneumonia in finisher pigs slaughtered during the winter months in a Federal abattoir [12]. Research by Tuovenin et al. detected seasonal variation in partial carcass condemnation data when using moving monthly averages [37] instead of quarterly rates as was used in our study. In terms of secular changes, results from our multi-level models using lung condemnation data predicted a decline across the study period in all regions. In 2001 there was an update made to the Code of Practice for the humane transport of farm animals [38], enforced by the CFIA and OMAFRA, whereby producers would be reported to the CFIA and penalized by issuance of an Administrative Monetary Penalty (AMP) if any pigs arriving at an abattoir exhibited severe signs of illness would be euthanized after reporting to the CFIA for (personal communication Ab Rehmtulla, DVM, OMAFRA, Stone Road, Guelph, Ontario). Perhaps the institution of monetary penalties lead to a decline in the number of pigs shipped to abattoirs showing signs of severe respiratory disease, compared to other less observable signs of disease that might be detected upon post-mortem inspection.

Higher rates of kidney condemnations in northern Ontario during the winter and fall compared to spring and summer, however, suggest that the incidence of nephritis in this colder area of the province may be influenced more by seasonal climactic variations compared to other regions in the province. We did not find a significant association between region and season for the lung condemnation data, and the predicted trends in lung condemnation rates did not coincide with the expected timeframes during which respiratory diseases were high in any of the agricultural regions in Ontario. Furthermore, there were no documented outbreaks of respiratory disease or diseases that cause nephritis in the province to explain the high rates of nephritis and pneumonia condemnations found in the data during 2001-2004. In contrast, the yearly trends in kidney nephritis condemnation rates that were predicted by the final model in eastern Ontario were consistent with expected increases in nephritis during the PCV-2 outbreak, and the third most likely space-time cluster, which coincided with the most likely spatial cluster for high kidney condemnation rates due to nephritis, took place in eastern Ontario during the timeframe in which the PCV-2 outbreak was known to have occurred. The decline in cases observed at the AHL by the spring of 2006, when the PCV-2 vaccine was introduced into swine herds in Ontario [28], coincides with the end of the third most likely space-time cluster of nephritis condemnations. Additionally, the decline in predicted rates of kidney condemnations due to nephritis in eastern Ontario from our multilevel models coincides with this cluster. Interestingly, very low rates of kidney condemnations were predicted in southern and western Ontario. There is no documented evidence to suggest lower rates of PCVAD took place in these regions during the outbreak.

Previous research [13,14], has demonstrated that regional differences influence condemnation data from provincially-inspected Ontario abattoirs. These differences may reflect different herd and farm densities, disease prevalence, and on farm management factors. It is also possible that plants in different regions may have different processing and inspection practices. Regardless of the reason for the spatial variation, by employing cluster detection methods that control for regional effects, it is possible to avoid these potential biases. For example, lung tissue from pigs is generally not processed for human consumption in provincial abattoirs, and at some abattoirs there may be less stringent recording of lung lesions in comparison with tissues that are important from a food safety and economic perspective. Furthermore, in plants where scalding tanks are used for de-hairing the pig carcass, lung tissue can become contaminated with scalding tank water and lung pathology can not consistently be evaluated (personal communication, Dr. Georges Branov, OMAFRA). Thus, clusters of high lung condemnation rates in space may represent different plant processing characteristics and/or reporting tendencies. The small spatial cluster of non-reporting of lung condemnations in southern Ontario may be related to the processing method (i.e. scalding tanks) used in abattoirs in this region, but we did not have information pertaining to individual abattoir de-hairing processes to further explore this as an underlying cause. Studies that investigate individual abattoir processing methods and their relation to condemnation information would be useful to improve our understanding of the extent that different methods bias condemnation data for disease surveillance.

To account for concerns regarding biasing factors that may be underlying the spatial clusters of high rates of kidney and lung condemnations, the space-time permutation model adjusts for purely spatial and temporal clusters that might exist in the data [39]. The third most likely space-time cluster of high kidney condemnation rates due to nephritis was detected during a time when the PCV-2 outbreak occurred in Ontario, and preceded temporal clusters of PCV-2 positive cases detected at the Animal Health Laboratory [27]. There were no significant spatio-temporal clusters of high rates of pneumonia found to predate or coincide with the timeframe during which there was widespread respiratory disease in finisher pigs in the province [1-6,40]. Temporal clusters of both kidney and lung condemnation categories did not correspond with the 2004-2006 outbreaks of PCV-2, swine influenza and PRRS. Since these temporal scans use data from all regions simultaneously, they are less sensitive at detecting a temporal change in a localized area.

Previous research that examined data collected from federally-inspected abattoirs supported the use of lung and kidney condemnation data for disease surveillance [12]. Because kidneys and lungs may be exported to countries that utilize these offal for pork products, this market difference, in addition to the requirement for licensed veterinary inspectors to oversee partial carcass condemnations in federally-inspected abattoirs, may result in improved data quality. However, federally-inspected abattoirs are large and there are only five that receive pigs from across the province of Ontario [12]. In contrast, provincially-inspected abattoirs are numerous and tend to be supplied by more local farms [13,14] making them more suited to the use of spatial and space-time surveillance methods. As a consequence of international trade restrictions, federal abattoirs in Canada may handle pigs that are, on average, of higher health status in comparison with provincial abattoirs, making provincial abattoirs more likely to detect disease outbreaks. Rates of condemnations related to pneumonia and nephritis were indeed higher than those found in an investigation of one Canadian federally-inspected abattoir [12], though the rates of pneumonia and nephritis in Ontario provincial abattoirs were lower on average than found in previously reported studies from other countries [30,31]. Without international standards for procedures and condemnation judgements at abattoirs, it is difficult to compare our results with those from other countries. While findings from our study are applicable to countries with well-developed meat inspection processes, they may be of particular interest for jurisdictions with numerous small abattoirs supplied by local farms [41].

While a number of statistical methods have been proposed and implemented in syndromic surveillance systems [42], each has advantages and limitations, and there is no clear consensus as to which are the most timely, sensitive and specific methods for analyses of various syndromic data [43]. An advantage of using the space-time scan statistic for cluster detection is that this method does not require the specification of baseline risk levels. However, in a prospective system, the specification of a reference time period is required, which may impact the sensitivity and specificity of cluster detection. The space-time permutation model is susceptible to population shift bias, whereby shifts in the background population distribution may create an interaction in space and time that is not related to increased disease risk [43]. The magnitude of this bias is generally a greater issue when investigating clusters in longer study periods, or numerous production cycles. In our study the 7-year timeframe during which some abattoirs closed across Ontario may have allowed population shift dynamics to bias the clusters that were detected. By shortening the length of time for our temporal and space-time scans and running multiple scans to cover the study period, the potential for population shift bias to impact results may be reduced. Another limitation of the scan statistic as it was applied in this study included the requirement to use circular scanning windows. Employing a scan statistic using flexibly shaped scanning windows may improve delineation of irregularly shaped clusters, such as those in irregularly shaped geographic locations [44] like the areas in Ontario that are bounded by the Great Lakes. However, for early outbreak detection, timeliness is most important and the circular-based scan performed better at cluster detection compared to a flexible scanning window during the early stages of an outbreak [44]. Applying scan statistics after adjusting for the effects of non-disease factors based on statistical models may improve upon cluster detection of true disease events [28,29]. Even sentinel-based surveillance systems should consider biases that could be corrected using these techniques.

Diseases in pigs such as PCV-2 and PRRS are often associated with co-infections involving a number of agents [1,2], pathogenesis is often multi-systemic [45-47], and pathological conditions such as nephritis can be caused by other primary agents, such as Leptospirosis [48] or porcine parvovirus (PPV) [26]. Specificity of gross pathological data are likely reduced when multiple etiologic agents can contribute to condemnations within the same syndrome category. However, the focus of a syndromic surveillance system is to detect changes in syndromes rather than a specific disease. Early warning based on less specific pre-diagnostic data are followed by more specific laboratory testing to determine the etiologic agents involved.

For any prospective disease surveillance system that uses data from slaughter plants, it is important to have traceability systems in place for rapid validation of clusters of high condemnation rates with farm-level and laboratory information. Canada is currently developing a system that will allow for the identification of all pig movements from farm to abattoir in the event of disease outbreaks [49]. In addition to traceability issues, there are other barriers to implementation of a syndromic surveillance system for pigs in Ontario at the present time. These include data privacy concerns, the need to integrate the data with existing disease surveillance systems, and practical issues pertaining to the collection, specialized training and personnel available for data collection. Any purposeful, standardized collection of data would need to be seamlessly integrated into procedures already in place at the abattoir. Development of timely validation and response procedures in the event of cluster detection is also required.

A prospective system that incorporates the purposeful, standardized collection of partial condemnation data pertaining to organs of interest for disease surveillance could be validated with other health information. The validity, sensitivity and specificity of these data for disease outbreak detection could thus be evaluated. An investigation is presently underway to assess whether whole carcass condemnations related to conditions including systemic sepsis, arthritis, and emaciation may be more appropriate for disease surveillance in this population of pigs.

## Conclusions

Based on our findings, condemnation information regarding lungs with pneumonia would not have provided useful data for detecting disease outbreaks in swine in the province of Ontario. Partial condemnation data pertaining to kidneys with nephritis did reflect the outbreaks more closely. This study identified non-disease factors that may bias partial carcass condemnation data for cluster detection. Applying cluster detection methods that control for influencing variables should be considered for their potential to improve the utility of abattoir data for disease surveillance.

## Authors' contributions

ALT performed the statistical analysis and drafted the manuscript. DLP, RMF and OB were involved in the conception and design, analysis and interpretation of data, and revising manuscript critically for important intellectual content. All authors read and approved the final manuscript.

## Supplementary Material

Additional file 1 **Table S1 Partial carcass condemnation reasons and frequencies in Ontario Provincial abattoirs (2001-2007)**. Reasons cited for partial carcass condemnations of market hogs in provincially-inspected abattoirs in Ontario from 2001-2007, total number of partial condemnations for all categories greater that 1%, and frequency of reason cited. Click here for file

Additional file 2 **Table S2 Univariable random intercept negative binomial models**. Modeling the association between lung pneumonia and kidney nephritis condemnation rates in provincial abattoirs in Ontario and year, season, census agricultural region, total pigs processed per year and median quarterly hog stock price, with abattoir as a random effect. Click here for file
