# Supplementary material for: Suitability and limitations of portion-specific abattoir data as part of an early warning system for emerging diseases of swine in Ontario
Source: BMC Vet Res. 2012 Jan 6;8:3. doi: 10.1186/1746-6148-8-3 (PMC3286412; doi:10.1186/1746-6148-8-3)
Supplement: Additional file 1 — Table S1 Partial carcass condemnation reasons and frequencies in Ontario Provincial abattoirs (2001-2007). Reasons cited for partial carcass condemnations of market hogs in provincially-inspected abattoirs in Ontario from 2001-2007, total number of partial condemnations for all categories greater that 1%, and frequency of reason cited. [file 1746-6148-8-3-S1.DOC]

**Additional file 1**

**Table s1 – Partial carcass condemnation reasons and frequencies in Ontario Provincial abattoirs (2001 – 2007)**

Reasons cited for partial carcass condemnations of market hogs in provincially-inspected abattoirs in Ontario from 2001 – 2007, total number of partial condemnations for all categories greater that 1%, and frequency of reason cited.

| **Condemnation Reason** | **Number** | **Percentage** |
| --- | --- | --- |
| **Elbow/hock arthritis** | 23045 | 3.55 |
| **Head/tongue abscess** | 12187 | 1.88 |
| **Kidneys cystic** | 47246 | 7.28 |
| **Kidneys nephritis** | 17790 | 2.74 |
| **Liver adhesion** | 25070 | 3.86 |
| **Liver parasitic** | 372689 | 57.42 |
| **Lungs pneumonia** | 24379 | 3.76 |
| **Skin dermatitis** | 9291 | 1.43 |
| **Skin parasitic** | 8389 | 1.29 |
| **Stifle joint arthritis** | 8558 | 1.32 |
