# Supplementary material for: Suitability and limitations of portion-specific abattoir data as part of an early warning system for emerging diseases of swine in Ontario
Source: BMC Vet Res. 2012 Jan 6;8:3. doi: 10.1186/1746-6148-8-3 (PMC3286412; doi:10.1186/1746-6148-8-3)
Supplement: Additional file 2 — Table S2 Univariable random intercept negative binomial models. Modeling the association between lung pneumonia and kidney nephritis condemnation rates in provincial abattoirs in Ontario and year, season, census agricultural region, total pigs processed per year and median quarterly hog stock price, with abattoir as a random effect. [file 1746-6148-8-3-S2.DOC]

**Additional File 2**

**Table s2 - Univariable random intercept negative binomial models**

Modeling the association between lung pneumonia and kidney nephritis condemnation rates in provincial abattoirs in Ontario and year, season, census agricultural region, total pigs processed per year and median quarterly hog stock price, with abattoir as a random effect.

**Lungs pneumonia condemnations Kidney nephritis condemnations**

| **Variable** | **IRR** | **P-value** | **95% CI** | **IRR** | **P-value** | **95% CI** |
| --- | --- | --- | --- | --- | --- | --- |
| **Year** |  |  |  |  |  |  |
| **2001** | referent |  |  | referent |  |  |
| **2002** | 0.74 | 0.01 | 0.58 – 0.93 | 1.64 | <0.001 | 1.32 – 2.05 |
| **2003** | 0.56 | <0.001 | 0.43 – 0.74 | 1.70 | <0.001 | 1.36 – 2.13 |
| **2004** | 0.40 | <0.001 | 0.29 – 0.53 | 1.03 | 0.82 | 0.81 – 1.31 |
| **2005** | 0.28 | <0.001 | 0.19 – 0.40 | 0.89 | 0.36 | 0.69 – 1.14 |
| **2006** | 0.36 | <0.001 | 0.26 – 0.51 | 0.96 | <0.001 | 0.75 – 1.23 |
| **2007** | 0.14 | <0.001 | 0.10 – 0.21 | 0.64 | <0.001 | 0.49 – 0.83 |
| **Season** |  |  |  |  |  |  |
| **Winter** | referent |  |  | referent |  |  |
| **Spring** | 0.84 | 0.16 | 0.67 – 1.05 | 0.84 | 0.04 | 0.71 – 0.99 |
| **Summer** | 0.76 | 0.03 | 0.60 – 0.98 | 0.70 | <0.001 | 0.59 – 0.83 |
| **Fall** | 0.75 | 0.02 | 0.59 – 0.95 | 0.72 | 0.02 | 0.61 – 0.86 |
| **Agricultural region** |  |  |  |  |  |  |
| **Central** | referent |  |  | referent |  |  |
| **Eastern** | 1.98 | 0.01 | 1.28 – 3.27 | 1.06 | 0.68 | 0.80 – 1.41 |
| **Northern** | 0.87 | 0.73 | 0.39 – 1.95 | 0.64 | 0.07 | 0.39 – 1.04 |
| **Southern** | 0.25 | <0.001 | 0.18 – 0.35 | 0.08 | <0.001 | 0.06 – 0.11 |
| **Western** | 0.73 | 0.06 | 0.53 – 1.02 | 0.04 | <0.001 | 0.03 – 0.05 |
| **# pigs processed/year*** | 0.48 | <0.001 | 0.45 – 0.51 | 0.99993 | <0.001 | 0.99992 –0.99994 |
| **Median quarterly price** | 1.01 | <0.001 | 1.007 – 1.014 | Not significant |  |  |

*modeled as log_10_total processed for lungs pneumonia model
